# Supplementary material for: Practice Changes in Checkpoint Inhibitor-Induced Immune-Related Adverse Event Management at a Tertiary Care Center
Source: Cancers (Basel). 2024 Jan 15;16(2):369. doi: 10.3390/cancers16020369 (PMC10814014; doi:10.3390/cancers16020369)

Supplementary Table S1: Demographic information for patients who developed immune-related adverse events in 2019 and 2021 by type of event, n=181

	No. (%)			
**Characteristic**	**Gastrointestinal, n=77**	**Pulmonary, n=76**	**Renal, n=15**	**Cardiac, n=13**
Median age (IQR), years	65 (54-72)	67.2 (58.1-72.7)	67 (62-73)	76 (76-78)
Median number of infusions (IQR)	4 (2-8)	5 (4-8)	4 (2-9)	2 (2-3)
Median duration of immunotherapy (IQR), days	73 (42-165)	84.5 (31.5-184.5)	82 (1-139)	21 (1-54.5)
Male	49 (64)	54 (71)	9 (60)	12 (92)
White race	72 (94)	63 (83)	14 (93)	10 (77)
Malignancy				
Melanoma	24 (31)	7 (9)	6 (40)	3 (23)
Genitourinary	21 (27)	18 (24)	3 (20)	3 (23)
Lung	10 (13)	29 (38)	3 (20)	4 (31)
Gastrointestinal	7 (9)	6 (8)	1 (7)	2 (15)
Head and neck	6 (8)	2 (3)	0 (0)	0 (0)
Other	9 (12)	14 (18)	2 (13)	1 (8)
Cancer stage				
I	2 (3)	1 (1)	0 (0)	1 (8)
II	3 (4)	5 (7)	0 (0)	3 (23)
III	14 (18)	11 (14)	5 (33)	0 (0)
IV	58 (75)	52 (68)	8 (53)	9 (69)
Unknown	0 (0)	7 (9)	2 (13)	0 (0)
Type of immunotherapy				
CTLA-4	11 (14)	2 (3)	0 (0)	0 (0)
PD-1/L1	34 (44)	51 (67)	9 (60)	8 (62)
Combination	32 (42)	23 (30)	6 (40)	5 (38)
Mortality in the study window[1]	23 (30)	38 (50)	2 (13)	5 (38)

[1]Follow-up window is 1 year after immune-related adverse event.

Supplementary Table S2: Immune-related adverse event Common Terminology Criteria for Adverse Events 5.0 grades in 2019 and 2021

	No. (%)		
**Event**	**2019**	**2021**	**P**
Diarrhea grade 2+	31 (81.6)	32 (82.1)	1.000
Colitis grade 2+	26 (56.5)	15 (48.4)	0.492
Pneumonitis grade 3+	30 (78.9)	22 (57.9)	0.048*

*Significant at the P<0.05 level (chi-square).

Supplementary Table S3: Gastrointestinal immune-related adverse event clinical management and outcomes by diarrhea grade in 2019 and 2021

	No. (%)			
	Diarrhea grade 1		Diarrhea grade >1	
**Outcome**	**2019, n=7**	**2021, n=7**	**2019, n=32**	**2021, n=31**
Median symptom duration (IQR), days	49 (4-90)	28 (21-36)	40 (20-93)*	23 (12-50)*
Outpatient consultation with a gastroenterologist[1]	4 (100)	4 (80)	19 (79)	12 (71%)
Any consultation with a gastroenterologist[2]	7 (100)	5 (71)	30 (94)	28 (90)
Median time to consultation with a gastroenterologist (IQR), days	15 (5-34)	26 (14-54)	21 (13-59)	15.5 (4.5-31)
Inpatient consultation with a gastroenterologist[3]	3/4 (75)	1/3 (33)	19/21 (90)	20/24 (83)
Follow-up with a gastroenterologist after discharge[3]	4/4 (100)*	0 (0)*	18/21 (86)	14/24 (58)
Median time to follow-up with a gastroenterologist after discharge (IQR), days[3]	12 (5-26)		24 (11-73)	31 (14-53)
Hospitalization	4 (57)	3 (43)	21 (66)	24 (77)
Median duration of hospitalization (IQR), days[3]	5 (4-9)	10 (9-23)	5 (3-8)	6 (3-10)
Endoscopy	6 (86)*	1 (14)*	30 (94)*	21 (68)*
Median time to endoscopy (IQR), days	4 (2-6)	17	8 (3-12)	8 (3-36)
Need for >1 steroid tapering attempt	6 (86)	4 (57)	9 (28)	3 (10)
Selective immunosuppressive therapy	3 (43)	2 (29)	25 (78)*	13 (42)*
Recurrence	2 (29)	0 (0)	15 (47)	10 (32)
Response	7 (100)	6 (86)	29 (91)	28 (90)
Readmission[3]	1/4 (25)	1/3 (33)	4/21 (19)	6/24 (25)

*Significantly different between 2019 and 2021 ($P<0.05$).

[1]39 patients presented with diarrhea prior to hospitalization (4 with grade 1 and 19 with grade >1 in 2019 and 4 with grade 1 and 12 with grade >1 in 2021).

[2]Includes as an outpatient before hospitalization, as an inpatient, or as an outpatient after hospital discharge.

[3]Among those hospitalized for grade 1 or grade >1 diarrhea in each period studied.

Supplementary Table S4: Gastrointestinal immune-related adverse event clinical management and outcomes by colitis grade in 2019 and 2021

	No. (%)			
	Colitis grade 0-1		Colitis grade >1	
**Outcome**	**2019, n=17**	**2021, n=19**	**2019, n=22**	**2021, n=19**
Median symptom duration (IQR), days	72 (25-104)	24 (12-36)	37 (18-51)	25 (14-51)
Outpatient consultation with a gastroenterologist[1]	13 (87)	10 (77)	10 (77)	6 (67)
Any consultation with a gastroenterologist[2]	16 (94)	17 (89)	21 (95)	16 (84)
Median time to consultation with a gastroenterologist (IQR), days	18.5 (12.5-40)	26 (13-50)	22 (13-42)	11 (3-24)
Inpatient consultation with a gastroenterologist[3]	7/8 (88)	7/11 (64)	15/17 (88)	14/16 (88)
Follow-up with a gastroenterologist after discharge[3]	8/8 (100)	8/11 (73)	14/17 (82)*	6/16 (38)*
Median time to follow-up with a gastroenterologist after discharge (IQR), days[3]	19 (10-49.5)	35 (14-67)	20 (7-73)	25.5 (14-44)
Hospitalization	8 (47)	11 (58)	17 (77)	16 (84)
Median duration of hospitalization (IQR), days[3]	4 (3-9)	3.5 (1-8)	5 (4-8)	9 (5-12.5)
Endoscopy	15 (88)*	10 (53)*	21 (95)*	12 (63)*
Median time to endoscopy (IQR), days	6 (3-10)	8 (2-36)	9 (2-14)	8 (2-27)
Need for >1 steroid tapering attempt	6 (35)	4 (21)	9 (41)	3 (16)
Selective immunosuppressive therapy	11 (65)	6 (32)	17 (77)	9 (47)
Recurrence	5 (29)	6 (32)	12 (55)	4 (21)
Response	16 (94)	16 (84)	20 (91)	18 (95)
Readmission[3]	0 (0)	3/11 (27)	5/17 (29)	4/16 (25)

*Significantly different between 2019 and 2021 ($P<0.05$).

[1]49 patients presented with colitis prior to hospitalization (13 with grade 0-1 and 10 with grade >1 in 2019 and 10 with grade 0-1 and 6 with grade >1 in 2021).

[2]Includes as an outpatient before hospitalization, as an inpatient, or as an outpatient after hospital discharge.

[3]Among those hospitalized for grade 0-1 or grade >1 colitis in each period studied.

Supplementary Table S5: Management outcomes in patients who received an outpatient consultation with a gastroenterologist and those who did not across both years

	No. (%)		
**Management outcome**	**Outpatient consultation, n=39**	**No outpatient consultation, n=11**	**P[1]**
Outpatient treatment	34 (87)	7 (64)	0.322
Hospitalization	19 (49)	7 (64)	0.496
Inpatient consultation with a gastroenterologist[1]	15/19 (79)	7/7 (100)	1.000
Follow-up with a gastroenterologist after discharge[1]	16/19 (84)	2 (29)	0.007
Endoscopic evaluation	32 (82)	5 (45)	0.023
Selective immunosuppressive therapy	23 (59)	6 (55)	1.000
Recurrence	15 (38)	5 (45)	0.723
Remission at final follow-up	36 (92)	9 (82)	0.214

[1]Diarrhea and colitis severity did not differ between the groups (P=0.662 and P=1.000, respectively).
[1]Among those hospitalized each group.

Supplementary Table S6: Univariate analysis of factors associated with various outcomes in gastrointestinal immune-related adverse events

Characteristic	Odds ratio	P
Recurrence		
Any consultation with a gastroenterologist[1]	3.3 (0.38-28.6)	0.276
Consultation with a gastroenterologist early vs late in immune-related adverse event disease course[2]	0.4 (0.1-0.9)	0.047*
Time from diagnosis to first consultation with a gastroenterologist	1.01 (1.00-1.03)	0.033*
Outpatient treatment		
Yes vs no	0.2 (0.1-0.6)	0.005*
Anti-diarrheal vs no treatment	0.3 (0.5-17.8)	0.209
Steroids vs no treatment	4.1 (0.8-22.0)	0.098
Anti-diarrheal vs steroids	0.7 (0.2-2.3)	0.613
Duration of initial steroid use	1.0 (1.0-1.0)	0.425
Selective immunosuppressive therapy	0.3 (0.1-0.8)	0.012*
Initial symptom improvement	0.04 (0.01-0.1)	<0.001*
Endoscopy	1.4 (0.5-4.4)	0.540
Readmission		
Outpatient consultation with a gastroenterologist	4.6 (0.9-22.3)	0.056
Outpatient treatment for gastrointestinal immune-related adverse event	0.55 (0.1-2.9)	0.487
Inpatient consultation with a gastroenterologist at first hospitalization	0.3 (0.1-1.4)	0.143
Consultation with a gastroenterologist early vs late in immune-related adverse event disease course[2]	0.2 (0.04-0.7)	0.012*
Time from diagnosis to first consultation with a gastroenterologist	1.0 (0.9-1.0)	0.193
Follow-up with a gastroenterologist after discharge (yes vs no)	2.1 (0.5-8.5)	0.282
Early follow-up with a gastroenterologist after discharge vs late follow-up[3]	0.8 (0.2-3.0)	0.781
Time to follow-up with a gastroenterologist after discharge	1.0 (0.9-1.0)	0.617
Duration of steroid use	1.0 (0.9-1.0)	0.232
Endoscopy	1.5 (0.3-7.5)	0.647
Need for >1 steroid tapering attempt		
Outpatient treatment for gastrointestinal immune-related adverse event	2.1 (0.7-6.0)	0.185
Consultation with a gastroenterologist early vs late in immune-related adverse event disease course[2]	0.3 (0.1-0.8)	0.024*
Duration of initial steroid use	1.02 (1.00-1.03)	0.025*

Duration of initial symptoms	1.02 (1.00-1.03)	0.018*
Sustained improvement of initial symptoms for >30 days	0.2 (0.1-0.5)	0.002*
Selective immunosuppressive therapy	3.4 (1.0-11.3)	0.048*
Endoscopy	2.9 (0.6-14.3)	0.201
Recurrence	6.7 (2.3-19.1)	<0.001*

*Statistically significant (P<0.05).

[1]Includes as an outpatient before hospitalization, as an inpatient, or as an outpatient after hospital discharge.

[2]Early consultation: <2 weeks before starting steroid therapy; late consultation: >2 weeks after starting steroid therapy.

[3]Early follow-up: <2 weeks after hospital discharge; late follow-up: >2 weeks after hospital discharge.

Supplementary Table S7: Pneumonitis clinical management and disease outcomes in 2019 and 2021 for peak pneumonitis grade 3+

	No. of new pneumonitis cases (%)		
**Outcome**	**2019, n=30**	**2021, n=22**	**P**
Outpatient consultation with a pulmonologist before hospitalization, n=4 in 2019 and n=1 in 2021	2 (50)	1 (100)	1.000
Any consultation with a pulmonologist[1]	29 (97)	22 (100)	1.000
Early consultation with a pulmonologist	27 (90)	22 (100)	0.253
Outpatient treatment for pneumonitis before hospitalization (n=4 in 2019, n=1 in 2021)	3 (75)	0 ()	0.400
Median time to consultation with a pulmonologist (IQR), days	1 (1-6)	2 (0.5-3.5)	0.898
Hospitalization	30 (100)	22 (100)	-
Median length of hospitalization (IQR), days	10 (6-14)	12 (6-13)	0.703
Inpatient consultation with a pulmonologist	26 (87)	21 (95)	0.381
Intensive care unit admission	14 (47)	10 (45)	0.329
Bronchoscopy	17 (57)	7 (32)	0.154
Median time to bronchoscopy (IQR), days	2 (1-9)	3 (2-10)	0.534
Follow-up with a pulmonologist after discharge	10 (33)	9 (41)	0.775
Follow-up with a pulmonologist within 1 month of discharge	4 (13)	5 (23)	0.637
Symptom improvement	13 (43)	9 (41)	1.000
Median duration of steroid use (IQR), days	30 (15-56)	32 (18-56)	0.795
Need for >1 steroid tapering course	12 (40)	5 (23)	0.233
Selective immunosuppressive therapy	7 (23)	3 (14)	0.488
Pneumonitis remission at final follow-up	12 (40)	15 (68)	1.000
Hospital readmission	14 (47)	5 (23)	0.049
Recurrence	15 (50)	10 (45)	0.785
Mortality	18 (60)	9 (41)	0.163

[1]Includes as an outpatient before hospitalization, as an inpatient, or as an outpatient after hospital discharge.

Suppl Figure S1

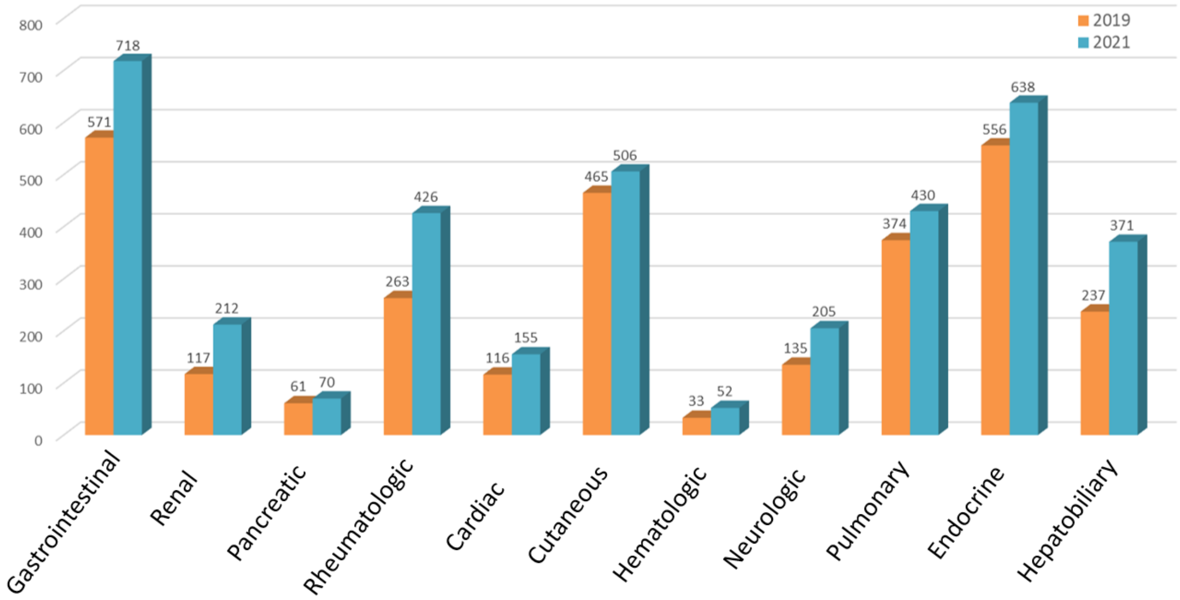

Suppl Figure S2

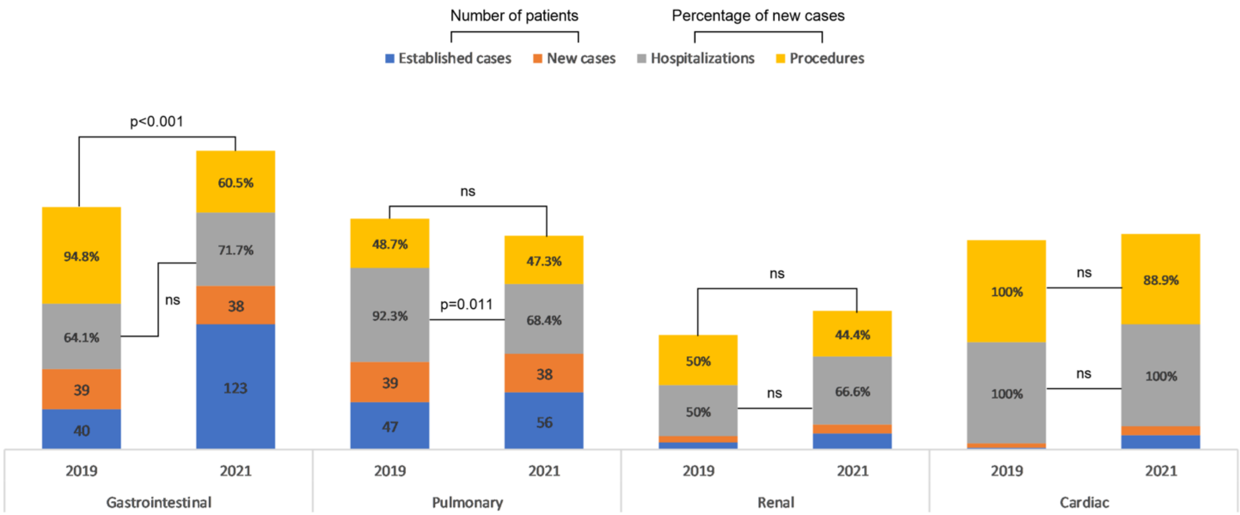

Suppl Figure S3a

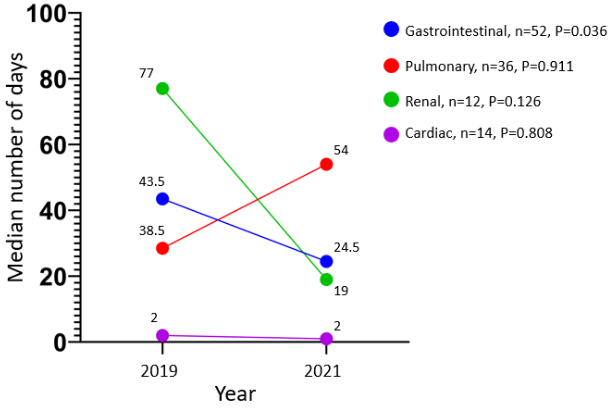

Suppl Figure S3b

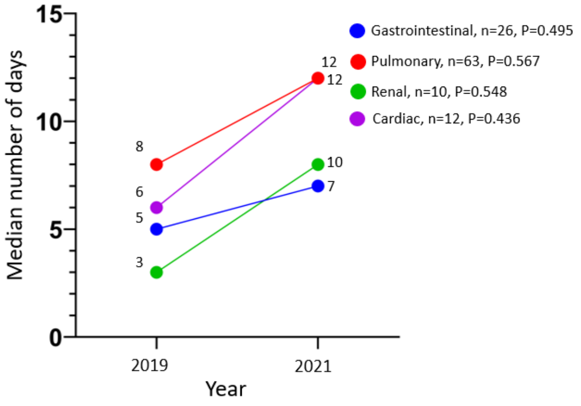

Suppl Figure S4a

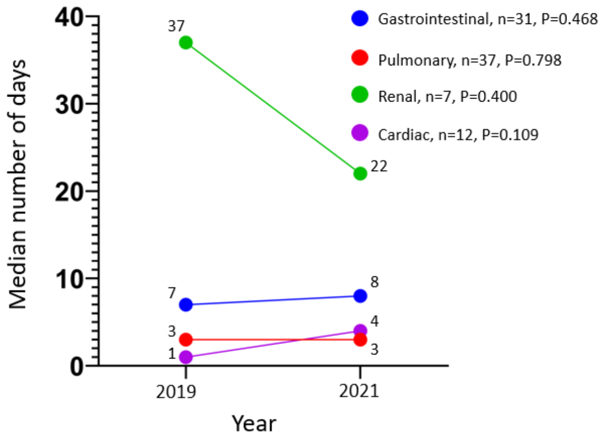

Suppl Figure S4b

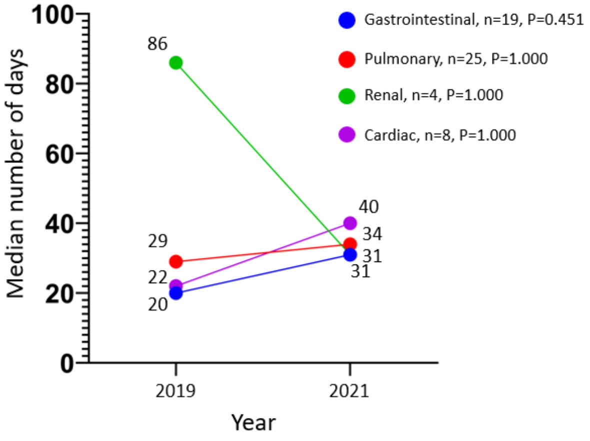

Supplement: Supplementary file 1 [file cancers-16-00369-s001.zip › cancers-2792396-supplementary.pdf]
